# Delivery of a Mental Health Intervention for Chronic Pain Through an Artificial Intelligence–Enabled App (Wysa): Protocol for a Prospective Pilot Study

**DOI:** 10.2196/36910

**Published:** 2022-03-31

**Authors:** Megha Gupta, Tanya Malik, Chaitali Sinha

**Affiliations:** 1 Wysa Inc Boston, MA United States

**Keywords:** chronic pain, AI-enabled mental health assistant, digital health intervention, mental health conversational agent, artificial intelligence, depression, mental health, anxiety, health care cost, conversational agent, chatbot, digital health

## Abstract

**Background:**

Patients with chronic pain often experience coexisting, long-term and debilitating mental health comorbidities such as depression and anxiety. Artificial intelligence–supported cognitive behavioral therapy (AI-CBT) interventions could offer cost-effective, accessible, and potentially effective resources to address this problem. However, there is not enough research conducted about the efficacy of AI-CBT interventions for chronic pain.

**Objective:**

This prospective cohort study aims to examine the efficacy and use of an AI-CBT intervention for chronic pain (Wysa for Chronic Pain app, Wysa Inc) using a conversational agent (with no human intervention). To the best of our knowledge, this is the first such study for chronic pain using a fully-automated, free-text–based conversational agent.

**Methods:**

Participants with self-reported chronic pain (n=500) will be recruited online on a rolling basis from April 2022 through posts on US-based internet communities within this prospective cohort. Informed consent is received from participants within the app, and the Wysa for Chronic Pain intervention is delivered remotely for 8 weeks. Outcome measures including a numeric pain rating scale and Patient-Reported Outcomes Measurement Information System–Pain Interference, Generalized Anxiety Disorder–7, and Patient Health Questionnaire–9 questionnaires administered to test the effectiveness of the intervention on reducing levels of pain interference, depression, and anxiety. The therapeutic alliance created with the conversational agent will be assessed through the Working Alliance Inventory–Short Revised instrument. Retention and use statistics will be observed for adherence and engagement.

**Results:**

The study will open for recruitment in April 2022, and data collection is expected to be completed by August 2022. The results for the primary outcomes are expected to be published by late 2022.

**Conclusions:**

Mental health conversational agents driven by artificial intelligence could be effective in helping patients with chronic pain learn to self-manage their pain and common comorbidities like depression and anxiety. The Wysa for Chronic Pain app is one such digital intervention that can potentially serve as a solution to the problems of affordability and scalability associated with interventions that include a human therapist. This prospective study examines the efficacy of the app as a treatment solution for chronic pain. It aims to inform future practices and digital mental health interventions for individuals with chronic pain.

**International Registered Report Identifier (IRRID):**

PRR1-10.2196/36910

## Introduction

### Background

Chronic pain is a long-term debilitating health concern that affects physical, psychological, cognitive, and social functioning [1-3] resulting in significant health care costs [4]. According to the American Academy of Pain Medicine, more than 1.5 billion people around the world have chronic pain [5]. At least 116 million US adults—more than the number affected by heart disease, diabetes, and cancer combined—live with common chronic pain conditions [6]. Research also suggests that 13% to 50% of adults in the United Kingdom are affected by chronic pain, with 10% to 14% experiencing moderate-to-severe disabling chronic pain [1,7]. The total health care cost of chronic pain ranges from $560 billion to $635 billion annually in the United States [8]. Chronic pain conditions are comorbid with depression, anxiety, sleep disturbances, fatigue, neurocognitive changes, and other symptoms [9].

### Chronic Pain and Mental Health

Mental health difficulties such as depression and anxiety are among the most common comorbidities present with chronic pain [10,11]. Between 20% to 50% of patients with chronic pain have comorbid depression [1,12], and patients with severe pain are more likely to be depressed [13]. Chronic pain often leads to patients being unable to focus on normal day-to-day activities and being constantly distracted by their pain, which is associated with poor mental health [14]. Moreover, patients experiencing from pain-related depression and anxiety are also likely to have worse outcomes from chronic pain [15,16]. Numerous studies have also suggested that individuals living with chronic pain are 2 to 3 times more susceptible to suicidal ideation and suicidal behaviors [17-19].

Similarly, research suggests that anxiety and fear arising from chronic pain are likely to interfere with recovery from the pain [15]. This has been observed with the development of maladaptive behaviors including catastrophizing [20], fear avoidance, and the reduction in helpful activities [21]. Chronic pain shares a bidirectional relationship with mental health [22], and the resolution of depression and anxiety are important components in the effective management of chronic pain.

### Pain Management

Chronic pain management through psychotherapeutic techniques is important since a commonly observed impact of chronic pain is the reduction in coherent behavior due to the consistent threat of pain [23]. Individuals report experiencing dissembling, with the pain moving beyond an acute experience and evolving into the prolonged state of a disabling disease [24].

Psychological treatments for chronic pain are usually cognitive or behavioral strategies aimed at reducing mental suffering and promoting active engagement with life [23]. They serve to overcome the pain perseverance paradox, which is the occurrence of self-defeating behaviors by those living with chronic pain [25]. Studies have demonstrated that treating mental health concerns such as depression can often result in better outcomes for treatment of pain [26]. Treatment strategies include behavioral techniques of relaxation, biofeedback, contingency management, exposure, and cognitive behavioral techniques that typically include programs with components of education, coping strategies training, and cognitive therapy [23].

Since depression in patients with chronic pain often goes unrecognized, it therefore remains untreated [27]. There is also a significant gap in the needs of patients with chronic pain and the care provided due to lack of therapeutic resources, acute stress on outpatient facilities [28], and worsening mental health experienced during the ongoing COVID-19 pandemic [29,30]. Several barriers to self-management of pain have been identified that include lack of support and resources, lack of time or fear of worsening the pain by engaging in physical activities, and difficulty in patient-physician interactions [31,32].

Research suggests that factors like support and encouragement from caregivers, access to a variety of self-management techniques, and treatment of depression can serve as facilitators in improving self-pain management [32]. Research also indicates the positive impact of a therapeutic alliance in improving the impact of treatment for individuals with chronic pain [33,34].

### Digital Mental Health Interventions for Chronic Pain

Digital mental health interventions can potentially address the issues of accessibility, rising health care costs, low availability of therapeutic care, and other barriers [35,36] associated with standard in-person treatments for chronic pain [37] and mental health [38,39]. Studies have reported that digitally delivered mental health interventions have a positive role to play in the self-management of depression and anxiety [40,41] and have been found effective in reducing the impact of chronic pain on the quality of life [42].

Currently, one way in which these digital mental health interventions address the barriers of accessibility is by augmenting digitally delivered tools with human coaches [43-46]. However, the success of this approach is dependent on the availability and degree of involvement of therapists, which influences the cost and scalability of such interventions. Another approach that can effectively tackle the problems of scalability and cost is an artificial intelligence (AI)-enabled conversational agent that mimics human dialog with users [47]. Health care interventions involving artificial intelligence–supported cognitive behavioral therapy (AI-CBT) conversational agents, aimed at reducing symptoms or improving self-management of mental health conditions and increasing mental well-being, are increasingly being used with positive outcomes [48-50]. Many of these conversational agent–based interventions have shown significant improvements on measures of common mental health problems like depression, psychological distress, anxiety, fear of heights, and loneliness [43,51-56]. Also, these studies suggest a positive correlation between engagement level with the conversational agent and reduction in psychological distress. However, there is a need for more evidence-based studies that focus on the efficacy of the interventions driven by AI-enabled conversational agents [57,58].

### Wysa for Chronic Pain

The intervention proposed in this study employs one such AI-CBT intervention, the Wysa for Chronic Pain app (Wysa Inc), that uses an AI-enabled conversational agent with a free-text conversational interface. It listens and responds to the user’s distress by recommending techniques and self-care tools based on CBT, behavioral reinforcement, and mindfulness, among other evidence-based therapies. Wysa supports the user in dealing with multiple challenges such as anxiety, sleep, low energy, motivation, loss, and pain. The app has been shown to be effective through mean improvements in symptoms of major depression (Patient Health Questionnaire–9 item [PHQ-9]) among users who were highly engaged with the app when compared to a low engagement group [59].

This is an exploratory study following a quantitative research design that aims to study the efficacy of a digital mental health intervention program for chronic pain when delivered solely by a conversational agent. The duration of the study is 8 weeks, and the efficacy will be calculated through the use of standardized statistical measures.

## Methods

### Overview

**Figure 1:**
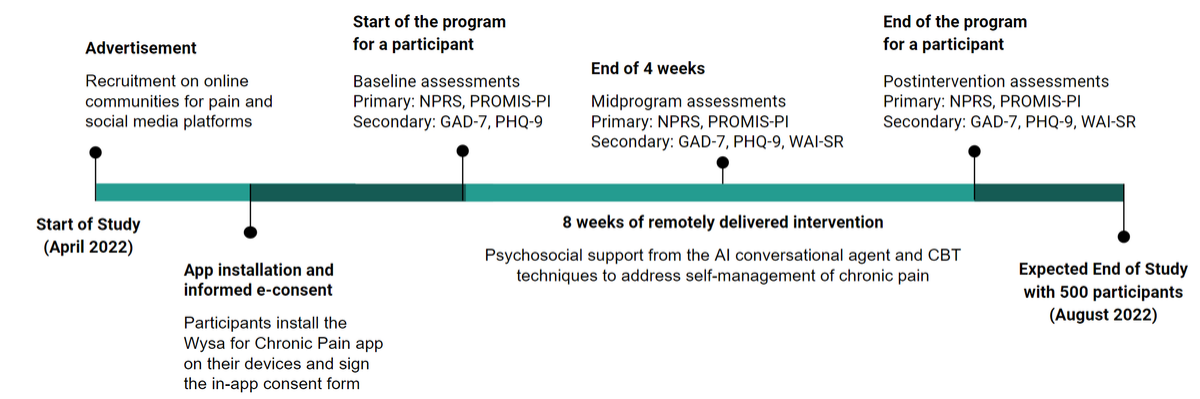
Timeline of the study. NPRS: numerical pain rating scale; PROMIS-PI: Patient-Reported Outcomes Measurement Information System Pain Inference; GAD-7: Generalized Anxiety Disorder, 7-item; PHQ-9: Patient Health Questionnaire, 9-item; WAI-SR: Working Alliance Inventory–Short Revised; CBT: cognitive behavioral therapy.

In this study, 500 individuals living with chronic pain will be recruited and administered an 8-week program on the Wysa for Chronic Pain app (a specific version of the publicly available Wysa app). This is an anonymous study where the participants enroll themselves by installing the app on their mobile phones and agree to participate in the study. We will restrict this version of the app to the Apple App Store and Google Playstore in the United States. Once enrolled in the program, participants will complete an assessment at the start of the study (comprising 4 measures), complete an 8-week program with the AI-led conversational agent during which another assessment (5 measures) will be taken halfway through the program, and complete the final set of assessments (5 measures) at the end of 8 weeks (Figure 1). Postintervention changes in pain interference will serve as the primary outcome for this study. Our secondary outcomes will include postintervention changes in depression and anxiety, as well as the therapeutic alliance between the user and conversational agent. The app will be free and available 24×7 to the participants.

### Screening

Eligibility criteria include being aged 18 years or older, experiencing chronic pain, and not receiving any form of professional mental health support. Individuals who do not self-report presenting with chronic pain will be excluded as a part of the recruitment criteria. The data of participants who score below 4 points on a 1-item numeric pain rating scale (NPRS) at the first assessment will be excluded from the analysis [60,61].

### Recruitment

Participants are currently being recruited on a rolling basis from US-based internet communities centered around the experiences of living with chronic pain. We will share information about the research study by posting on social media platforms. The purpose of these posts is to introduce potential participants to the Wysa for Chronic Pain app, inform them of the aims of this research study, and subsequently invite them to download the app on their devices. All participants will need to have an Android or iOS phone and have access to the internet while using the app. Interested participants will be screened on the basis of self-reported scores on the NPRS and their responses to a set of questions that will collect baseline demographics (age range), type of chronic pain, and the time since onset.

### Ethics

Participants will remain anonymous for the duration of the study and are being recruited from a US-only population. After participants download the Wysa for Chronic Pain app and agree to the app’s Terms of Service and Privacy Policy, they will opt in to the study using a consent screen following Wysa’s security and compliance guidelines. The consent screen states the purpose of the trial, potential risks and benefits associated with participation in the trial study, and mechanism for opting out of the study at any time. They will also be informed of the ways in which the usage data generated from their participation in the study could potentially be used.

### Intervention

**Figure 2:**
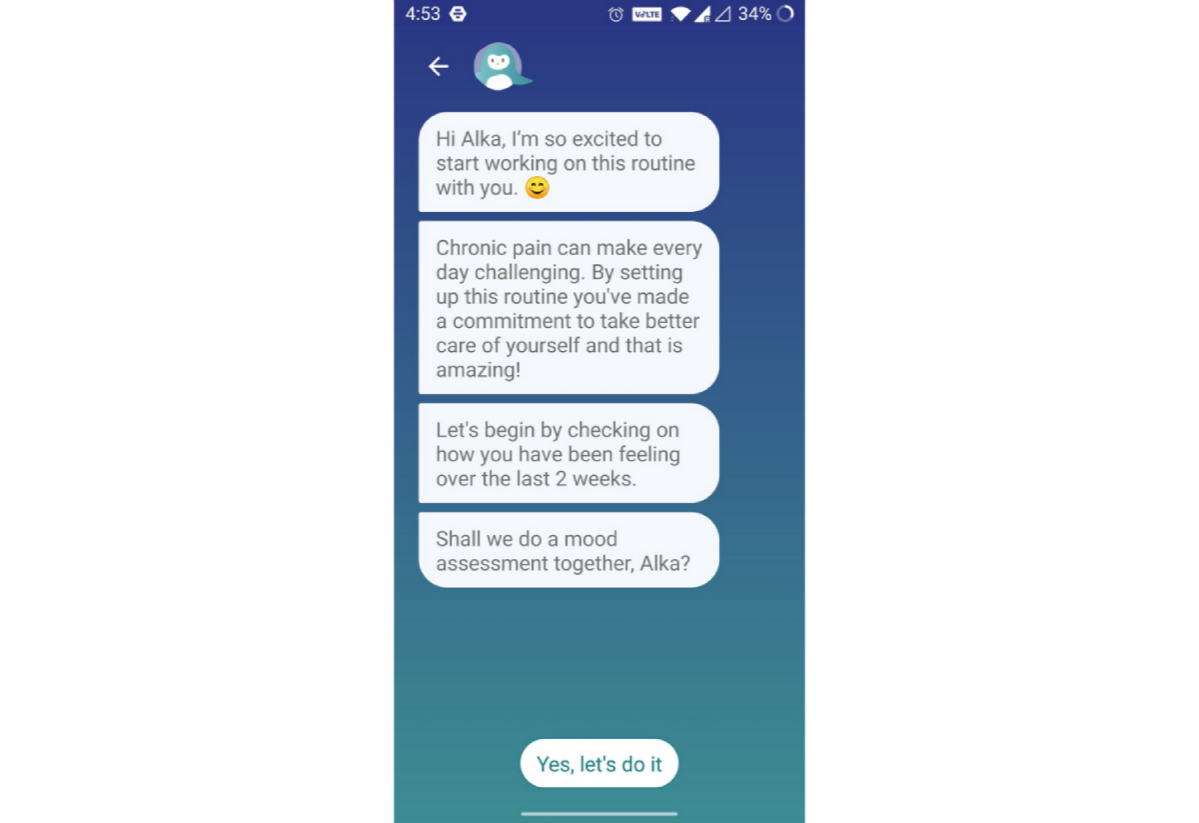
Onboarding conversation with the conversational agent that sets the expectation for the program and administers the first assessment.

A separate version of the publicly available Wysa app, Wysa for Chronic Pain, is being used for this study. This is a conversational agent–only version (ie, it does not include one-on-one human coach support, which is an option with the publicly available app). The AI-enabled free-text conversational agent offers participants a space to talk about their pain, depression, anxiety, and other issues arising from disturbances due to chronic pain. The conversational agent acts as a companion in their journey (Figure 2) of learning to live with pain and helps them build resilience by guiding them through self-care tools based on CBT and other techniques. The goal of the intervention is to improve the quality of life for the user as their ability to manage the pain improves.

**Figure 3:**
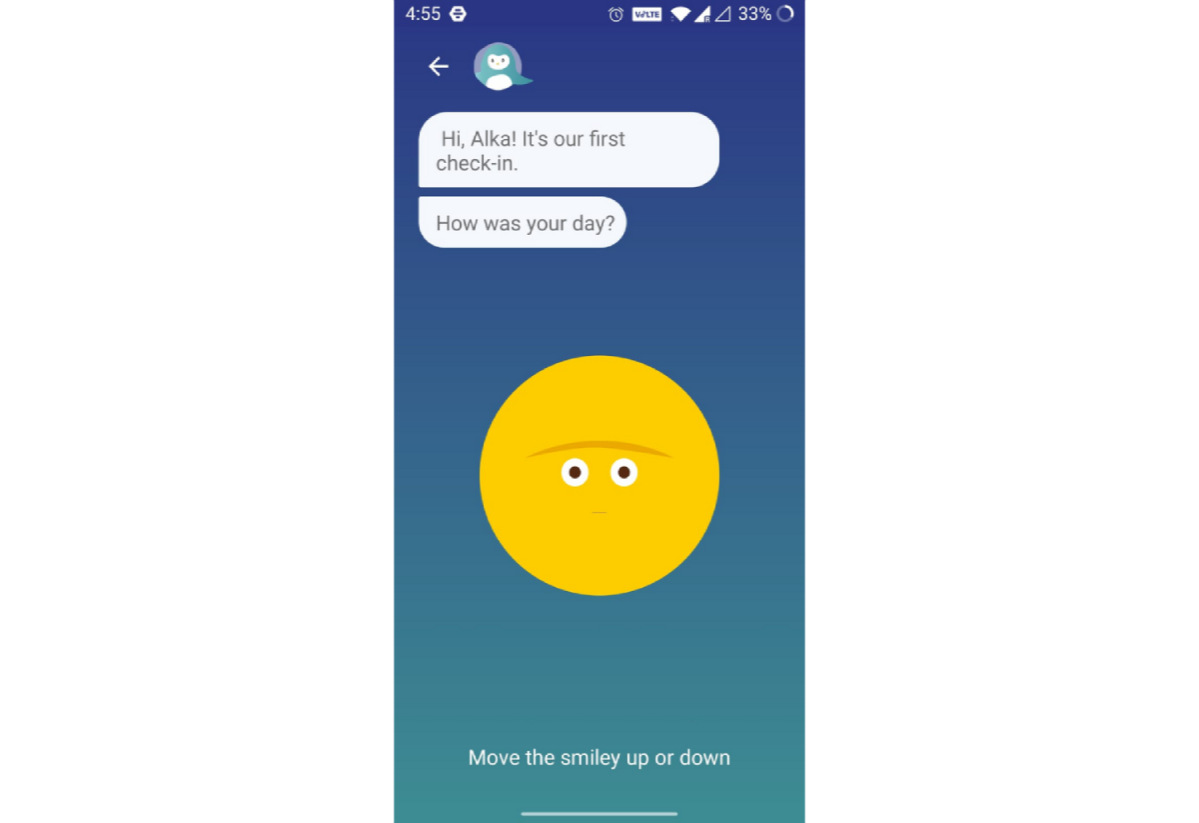
First mood check-in with the conversational agent.

The conversational agent builds an 8-week road map for the user and does daily morning and evening check-ins to encourage adherence to the program (Figure 3). Users are encouraged to set a daily goal for themselves based on what gives them joy, and ideas are suggested to help them engage in these activities, even if in a small way. Completing these activities on a regular basis gives the users a sense of joy and achievement and fosters the belief that they are capable of simultaneously managing their pain and living a normal life [62,63].

Steps included in the user’s journey includes participating in activities that comprise a mix of positive psychology, acceptance and commitment therapy, and CBT techniques that will be conducted in an organized, weekly fashion to encourage the development of skills aimed at coping with pain and the reduction of depression and anxiety symptoms.

In contrast to traditional worksheets or psychoeducation materials common in digital mental health [64,65], all therapeutic interventions in this study are conducted in a conversational format via the Wysa AI-enabled agent, which guides the user through the program in order to create a sense of support similar to in-person therapy [66]. The conversational nature of the interaction is meant to engage the user more, thereby increasing adherence to the effective intervention for chronic pain. The AI-enabled conversational agent provides empathetic support to the user, answers their questions, and guides them through uncertainty and a potential lack of trust in the bot as they proceed with their journey [67]. A rapport is built between the user and Wysa by the conversational agent’s ability to add personalized elements to the conversation, including welcoming messages, addressing the user by the nickname chosen by them during onboarding, and providing clarifying examples. All these are important elements of a therapeutic conversation [68].

**Figure 4:**
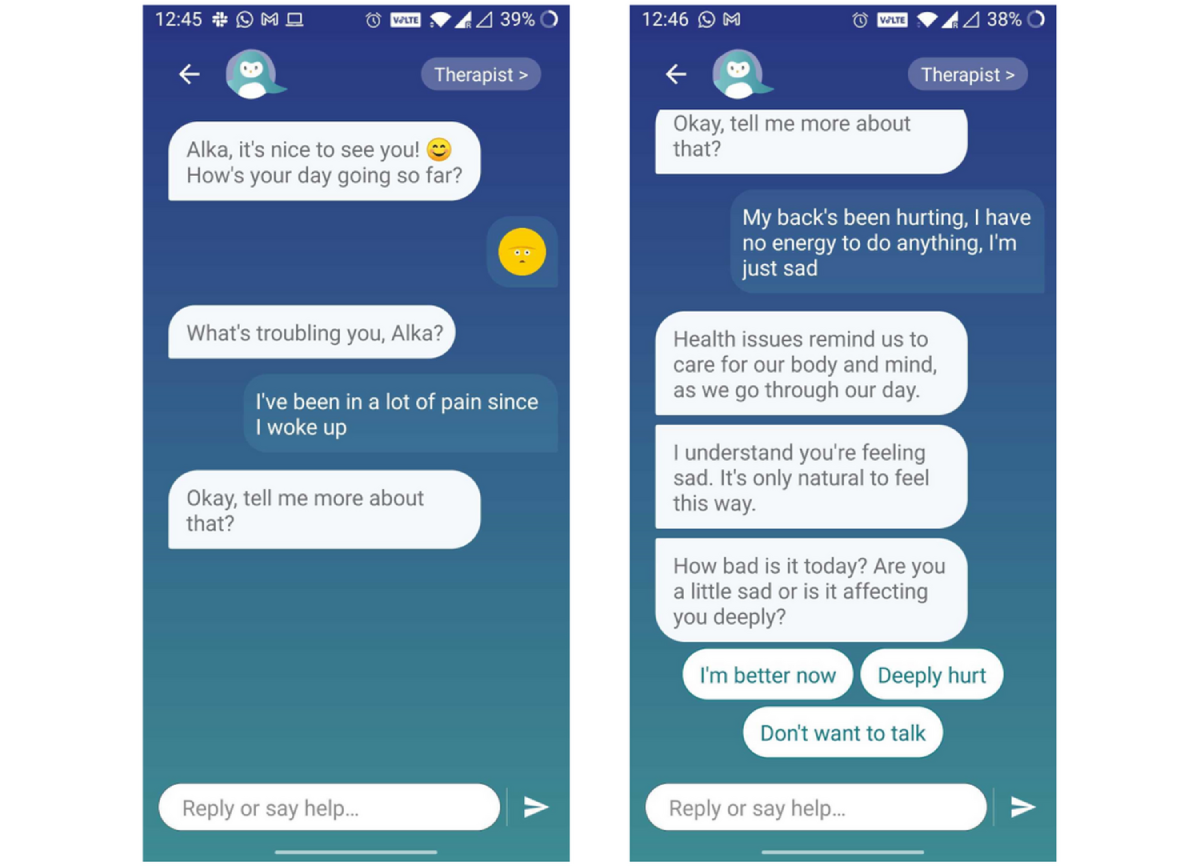
Example screenshots showing the conversational style of the app and the flexibility given to the user through free-text inputs.

The app provides the user an open space for conversation by giving the user the ability to converse with the conversational agent through free text (Figure 4). The free-text input enables flexibility and genuineness in the conversation, and allows the user to speak their mind. The app uses natural language processing, natural language understanding, and machine learning to interpret the user’s messages, and provides the user with supportive listening and appropriate responses to unique situations using interventive techniques, thus creating an efficient interactive environment [55,69]. The conversational agent provides users with empathetic support in a nonjudgmental environment, an important aspect of health care [70]. The AI uses custom models trained by data scientists, clinicians, and conversation designers that detect different aspects of what the user is conveying [71].

### Primary Outcome Measure

Study participants will complete the self-administered measures, NPRS and the Patient-Reported Outcomes Measurement Information System–Pain Inference (PROMIS-PI) short form 6b, at the baseline, midpoint, and after the completion of the study. Both measures will be administered through the Wysa app and will be completed on the participants’ own devices.

#### Numeric Pain Rating Scale

The NPRS is a unidimensional measure of pain intensity in adults with chronic pain [72]. It is a segmented 11-point numeric scale in which the respondent rates the intensity of their pain on an integer scale of 0 (no pain) to 10 (worst pain imaginable). The minimal clinically important change score is taken to be a 2 point reduction [73,74].

#### Patient-Reported Outcomes Measurement Information System–Pain Interference

The 6-item PROMIS-PI was developed by the National Institutes of Health to measure the negative effects of pain on physical, mental, cognitive, emotional, social, and recreational functioning. This study uses a 6-item pain interference scale that aims to measure the extent to which pain causes a hindrance in various aspects of an individual’s life, including those related to activities done for fun as well as day-to-day activities. A 5-point ordinal rating scale is used with options being 1 (not at all), 2 (a little bit), 3 (somewhat), 4 (quite a bit), and 5 (very much). The raw scores are converted to T-scores based on a scoring manual. Although the PROMIS-PI forms are relatively new, their responsiveness has been found to be comparable to legacy pain measures [75]. A reduction in PROMIS-PI scores at the end of the study will indicate a positive impact of the intervention. Minimal clinically important change score is defined as at least 2.0 points for pain interference [76].

### Secondary Outcome Measures

In order to observe ancillary impacts of the intervention, study participants will complete 2 additional self-administered measures (PHQ-9 and Generalized Anxiety Disorder, 7-item [GAD-7]) at the baseline, midpoint, and after the completion of the study. The Working Alliance Inventory–Short Revised (WAI-SR) will be administered at the midpoint and after completion of the study only. All measures will be administered through the Wysa app itself and will be completed on the participants’ own devices.

#### Generalized Anxiety Disorder

GAD-7 is a widely adopted, standardized, and validated 7-item self-report questionnaire developed to assess symptoms of generalized anxiety disorder. This 7-item anxiety scale has been found to have good reliability as well as construct, procedural, and factorial validity [77]. This assessment asks the participants to assess themselves over the last 2 weeks with questions about whether they felt nervous or anxious, had trouble relaxing, became easily annoyed or irritated, etc. The response categories include 0 (not at all), 1 (several days), 2 (more than half the days), and 3 (nearly every day). The final score is calculated by summing over the 7 questions, and scores of 5, 10, and 15 are taken as the cutoff points for mild, moderate, and severe anxiety, respectively. A reduction in the GAD-7 score at the end of the study will indicate a positive impact of the intervention. Clinically meaningful effect size is defined as at least 4.0 points for the GAD-7 [78].

#### Patient Health Questionnaire

PHQ-9 is a widely used, standardized, validated 9-item survey assessment tool for depression that has been shown to be valid and reliable as a longitudinal clinical tool [79]. It has demonstrated high criterion validity among populations with high rates of physical distress and has been found to be valid for diverse modalities of administration, including those via touchscreens [80]. This assessment asks the participants to assess themselves over the last 2 weeks with questions about whether they felt pleasure in doing things, felt tired or having little energy, had thoughts of hurting themselves, etc. The response categories and associated numerical scores are identical to that of GAD-7. The overall score is calculated by summing over the 9 questions and is used to monitor the severity of depression and response to treatment. A reduction in the PHQ-9 scores at the end of the study will indicate a positive impact of the intervention. Clinically meaningful effect size is defined as at least 5.0 points for the PHQ-9 [79].

#### Working Alliance Inventory–Short Revised

The WAI-SR is a well-established measure of therapeutic alliance, consisting of a total score and 3 subscales: bond, goal, and task [81]. This questionnaire asks the participants 12 questions related to whether the therapy gave them new ways of looking at their problem, whether their therapist cares for and respects them, and whether they agree with their therapist on the goals set for them. The response categories lie on a 5-point Likert scale and include 1 (seldom), 2 (sometime), 3 (often), 4 (very often), and 5 (always). Ratings are summed at the end, with a higher total indicating better therapeutic alliance. The WAI-SR will be administered at the end of the study via the app’s conversational interface, in which the word therapist will be changed to Wysa. This measure demonstrates high internal consistency (Cronbach α>.90) [82]. Mean total scores of 3.59 (out of 5) are considered high [83].

### Statistical Analysis

We will use the Wilcoxon signed-rank test to measure the efficacy of the intervention by comparing baseline and postintervention assessment scores on the NPRS, GAD-7, and PHQ-9 scales. The Wilcoxon signed-rank test is a nonparametric statistical hypothesis test used to compare the differences between 2 populations using a set of matched samples. We will also use the Wilcoxon signed-rank test for measuring the median of therapeutic alliance using the WAI-SR scale. We chose this test because we believe that our data will not be normally distributed, in which case a nonparametric test is more suitable. Finally, we will do a paired *t* test for measuring if the intervention resulted in any significant changes on the PROMIS-PI scale since its scores are mapped to T-scores. Rolling and ongoing recruitment will mediate any potential concerns regarding the power of the study and the multiple testing problem where Type I errors may get inflated.

## Results

Recruitment for this study will begin in April 2022 and continue on a rolling basis until 500 participants have been recruited. The baseline, midpoint, and postintervention assessment data for participants who have completed their 8-week intervention will be evaluated to determine if the intervention had any significant effect on their pain interference, depression, and anxiety scales. Although participants are encouraged to check in twice a day, there is no mandate for continuation in the program. Final outcomes will be based on end of program assessments, and engagement is evaluated across the cohort to evaluate efficacy in a real-world use setting. Another round of recruitment may be needed if the retention rate is low. App use, engagement, and retention will also be assessed to determine the acceptability of an AI-only conversational agent to support chronic pain management. From this pilot, we hope to learn more about what frequency and how many check-ins can be considered as sufficient use of the app so as to have desirable outcomes for the user. Data collection for all participants is expected to be completed by August 2022 and results to be published by late 2022.

## Discussion

### Summary

To the best of our knowledge, this is the first study to assess the efficacy of a fully automated, free-text–based conversational agent as an assistant for chronic pain patients. Prior to this, Hauser-Ulrich et al [84] evaluated the efficacy and acceptance of the Smartphone-Based Health Care Chatbot to Promote Self-Management of Chronic Pain (SELMA) in pain self-management for chronic pain patients. Some of the shortcomings of SELMA, as pointed out by its users, were the conversation being too structured, there not being enough answer options, and no ability to write free text. In addition, the notifications were sent at a fixed time and there was no way to personalize the app.

Wysa for Chronic Pain overcomes these shortcomings. Moreover, apart from using a conversational flow tailored for chronic pain, it also provides participants with a wide array of self-care tools they can use to deal with other issues like insomnia, depression, anxiety, and negative thoughts anytime they want [59,85,86]. Wysa for Chronic Pain has proven high engagement and efficacy when the intervention uses a conversational agent enhanced by a human coach [87]. This study aims to assess the efficacy of this intervention using the conversational agent alone. The quality of the therapeutic alliance built between the user and the conversational agent will also be assessed, which will further inform the utility of digital mental health interventions.

Treatment of chronic pain often requires a multidisciplinary and multidimensional approach, targeting both the physical and psychological aspects of the disease. Many techniques like behavioral activation, mindfulness exercises, CBT, interpersonal psychotherapy, and psychoeducational tools have been tried for different kinds of pain, with each showing promise for different aspects of pain management [88]. This is one of the reasons why digital health interventions are becoming increasingly popular as they remove the barriers of experienced disrespect, distrust, and dismissal encountered while seeking care for chronic pain [89]. There have been several efforts at building digital health interventions for chronic pain and studying their efficacy for various kinds of patient groups. While many have shown promising results for different aspects of pain management, most of them involved human therapists [45,46], focused on passive consumption of psychoeducational content [45,90], or required an additional device [91,92]. Any requirement of a therapist or an additional device greatly limits the scalability and accessibility of a digital mental health intervention, and passive consumption of psychoeducational content isn’t interactive, and hence, may not be very engaging [57].

To address these challenges, this study proposes to investigate the efficacy of a conversational agent–led intervention for chronic pain without the need for human coaches or special devices. The Wysa for Chronic Pain app involves no human coach in the loop, but the free-text–based AI-enabled conversational agent is capable of understanding and responding to a user’s messages like a human therapist would. The advantages of using a digital mental health conversational agent like Wysa are manifold. First, therapy and self-care tools grounded in CBT, mindfulness, and other evidence-based therapies become immediately accessible to the patient via their mobile phone. Second, the strain on human resources is reduced. Third, anonymity allows patients to share their thoughts and feelings more freely, thereby increasing alliance. Fourth, especially in the context of chronic pain, when even routine activities seem overwhelming, a mobile assistant puts the resources literally in the user’s hands and takes away the effort, stress, and additional challenges required to go meet a therapist. Finally, the conversational agent also acts like a companion who is always there to talk to [93].

Another facet of the treatment for chronic pain includes the therapeutic alliance between the individual and the support system. Emerging evidence for chronic musculoskeletal pain indicates that a strong therapeutic alliance may improve pain outcomes [94]. Therapeutic alliance was consistently a predictor of outcome in a study with 182 patients with lower back pain that assessed function, global perceived effect of treatment, pain, and disability [95]. Another study showed that enhanced therapeutic alliance combined with treatment led to clinically meaningful improvements in pain intensity and muscle pain sensitivity in patients with chronic low back pain [96]. This study looks at the levels of therapeutic alliance formed with the AI-enabled conversational agent and the impact on treatment outcomes for the participants.

### Strengths and Limitations

A challenge associated with most digital mental health interventions is ensuring adherence and engagement [90]. Adherence is key to benefiting from a self-management program. To address this potential problem and promote adherence, the app has been built using the principles of behavioral activation, which are supported conversationally and have specific customizations for chronic pain [87].

To encourage participation, the study has intentionally been structured to be anonymous. The app does not require the creation of an account nor does it ask for personal identification data (name, age, location, etc). Any sensitive information, if provided accidentally by a participant in a conversation, is identified and redacted by an algorithm to prevent retention in the system. This kind of anonymity may promote trust in the app among the users and encourage them to feel more open and engage with the conversational agent. It is only available in English, which is a limitation of the service.

Although recruitment for this study is being done from online communities of people living with chronic pain, one challenge that remains is the inability to verify chronic pain in the cohort recruited. All participants will be self-referred, thus making it difficult to assess whether they satisfy the inclusion criteria. In addition, their presence in online communities oriented to the support of pain may indicate sampling bias toward individuals with a greater willingness to learn about pain self-management [97]. Another potential limitation of this study may be the self-reported nature of the assessments, which may indicate subjective efficaciousness due to the absence of physician-interventive metrics for change. Self-reporting also involves the risk of random answers offered by some participants. We will examine the data for outliers (any data points that lie 2 to 3 standard deviations away from the mean) and use an appropriate statistical methodology to improve data quality.

### Conclusions

Chronic pain is one of the leading causes of long-term disability in the world. It is a complex problem with roots in social, economic, physical, and psychological aspects and affects more than 1.5 billion people worldwide. Treatment is difficult, and it often requires continuous pain management. Depression and anxiety are the most common comorbidities occurring with chronic pain, and psychological interventions have been shown to result in better outcomes for treatment of chronic pain. Digital mental health solutions make these interventions accessible and affordable. This study describes a novel AI-CBT intervention, the Wysa for Chronic Pain app, which is completely led by a free-text–based, AI-enabled conversational agent. The intervention takes the user through a variety of techniques and self-care tools grounded in CBT and mindfulness, which enable the user to learn how to self-manage their pain on a regular basis. The results from this study will be important in understanding the efficacy of such an intervention that can potentially serve as a scalable and cost-effective resource for chronic pain patients around the globe.

## Abbreviations

AI: artificial intelligence
AI-CBT: artificial intelligence–supported cognitive behavioral therapy
CBT: cognitive behavioral therapy
GAD-7: Generalized Anxiety Disorder, 7-item
NPRS: numerical pain rating scale
PHQ-9: Patient Health Questionnaire, 9-item
PROMIS PI: Patient-Reported Outcomes Measurement Information System–Pain Inference short form 6b
SELMA: Smartphone-Based Health Care Chatbot to Promote Self-Management of Chronic Pain
WAI-SR: Working Alliance Inventory–Short Revised
